# Genome assembly of the basket willow, *Salix viminalis*, reveals earliest stages of sex chromosome expansion

**DOI:** 10.1186/s12915-020-00808-1

**Published:** 2020-06-30

**Authors:** Pedro Almeida, Estelle Proux-Wera, Allison Churcher, Lucile Soler, Jacques Dainat, Pascal Pucholt, Jessica Nordlund, Tom Martin, Ann-Christin Rönnberg-Wästljung, Björn Nystedt, Sofia Berlin, Judith E. Mank

**Affiliations:** 1 Department of Genetics, Evolution & Environment, University College London, London, UK; 2 Department of Biochemistry and Biophysics, National Bioinformatics Infrastructure Sweden, Science for Life Laboratory, Stockholm University, Stockholm, Sweden; 3 Department of Molecular Biology, National Bioinformatics Infrastructure Sweden, Science for Life Laboratory, Umeå University, Umeå, Sweden; 4 Department of Medical Biochemistry and Microbiology, National Bioinformatics Infrastructure Sweden, Science for Life Laboratory, Uppsala University, Uppsala, Sweden; 5 Department of Medical Sciences, Section of Rheumatology, Uppsala University, Uppsala, Sweden; 6 Department of Plant Biology, Uppsala BioCenter, Linnean Centre for Plant Biology, Swedish University of Agricultural Sciences, Uppsala, Sweden; 7 Department of Medical Sciences, National Genomics Infrastructure, Science for Life Laboratory, Uppsala University, Uppsala, Sweden; 8 Department of Medical Sciences, Science for Life Laboratory, Uppsala University, Uppsala, Sweden; 9 Department of Cell and Molecular Biology, National Bioinformatics Infrastructure Sweden, Science for Life Laboratory, Uppsala University, Uppsala, Sweden; 10 Department of Zoology and Biodiversity Research Centre, University of British Columbia, Vancouver, Canada

**Keywords:** Sex chromosomes, W-chromosome, Recombination suppression, Willow, Salix

## Abstract

**Background:**

Sex chromosomes have evolved independently multiple times in eukaryotes and are therefore considered a prime example of convergent genome evolution. Sex chromosomes are known to emerge after recombination is halted between a homologous pair of chromosomes, and this leads to a range of non-adaptive modifications causing gradual degeneration and gene loss on the sex-limited chromosome. However, the proximal causes of recombination suppression and the pace at which degeneration subsequently occurs remain unclear.

**Results:**

Here, we use long- and short-read single-molecule sequencing approaches to assemble and annotate a draft genome of the basket willow, *Salix viminalis*, a species with a female heterogametic system at the earliest stages of sex chromosome emergence. Our single-molecule approach allowed us to phase the emerging Z and W haplotypes in a female, and we detected very low levels of Z/W single-nucleotide divergence in the non-recombining region. Linked-read sequencing of the same female and an additional male (ZZ) revealed the presence of two evolutionary strata supported by both divergence between the Z and W haplotypes and by haplotype phylogenetic trees. Gene order is still largely conserved between the Z and W homologs, although the W-linked region contains genes involved in cytokinin signaling regulation that are not syntenic with the Z homolog. Furthermore, we find no support across multiple lines of evidence for inversions, which have long been assumed to halt recombination between the sex chromosomes.

**Conclusions:**

Our data suggest that selection against recombination is a more gradual process at the earliest stages of sex chromosome formation than would be expected from an inversion and may result instead from the accumulation of transposable elements. Our results present a cohesive understanding of the earliest genomic consequences of recombination suppression as well as valuable insights into the initial stages of sex chromosome formation and regulation of sex differentiation.

## Background

Sex chromosomes, genomic regions associated with either males or females, have evolved independently many times in the eukaryotes [1, 2]. Sex chromosomes come in two general forms in organisms where sex is expressed in the diploid phase of the life cycle. X-Y sex chromosomes form where the sex-specific Y chromosome is associated with males (male heterogamety), and Z-W sex chromosomes form where the sex-specific W chromosome is associated with females (female heterogamety). Both of these sex chromosome types emerge after recombination is halted between a homologous pair of chromosomes [3, 4], which allows the X and Y or Z and W chromosomes to diverge from each other. Studies in systems with highly diverged, independently evolved sex chromosomes have revealed many shared genomic properties across a broad array of taxa [1, 2, 5], and sex chromosomes therefore represent an important example of convergent genome evolution.

In addition to promoting sex chromosomes’ divergence from one another, recombination arrest in the sex-determining region (SDR), the region harboring the sex-determining locus, leads to a range of non-adaptive consequences for the sex-limited Y or W chromosome. These include the build-up of deleterious variation and repetitive elements, as well as the loss of gene activity [6–8]. Due to the longstanding focus on systems with highly divergent sex chromosomes, the speed and order at which these processes occur after recombination suppression remain largely unclear.

Additionally, over evolutionary time, the non-recombining region can expand, resulting in strata or regions with differing levels of divergence between the X and Y or Z and W chromosomes [9–13]. Broadly defined, a stratum is a contiguous region along the sex chromosome where recombination suppression occurred at roughly the same time. In a nascent stratum, recombination may still occur occasionally, but the sex chromosomes are nonetheless in the earliest stages of divergence. Most importantly, although there will be substantial variance in divergence, repetitive element accumulation, and transcriptional loss, there will be more similarity for these traits within a stratum than among different strata or the pseudo-autosomal regions. Multiple strata can occur, resulting in regions along the sex chromosome with different levels of divergence [10, 14, 15].

Expansion of the non-recombining region and the emergence of new strata may occur gradually, in which case we might expect only partial recombination suppression in the youngest stratum, in conjunction with substantial heterogeneity in X-Y or Z-W divergence [16–19]. Alternatively, some have suggested that strata form instantaneously, via large-scale inversions on the Y or W chromosome [20], which prevent recombination between the sex chromosomes along the entirety of the reversed region.

The answers to these questions have important implications beyond sex chromosomes. Halting recombination permanently links co-adapted gene complexes [21–24], also referred to as supergenes. Y and W chromosomes are thought to represent sex-specific supergenes, linking loci with sex-benefit alleles to the sex-determining locus [25–28]. Supergenes have resurfaced recently as a major potential adaptive mechanism [29–33], and in so doing have implicated recombination suppression as a crucial component of complex phenotypic adaptation. However, it is important to note that alternative, non-adaptive mechanisms have been suggested for recombination suppression on sex chromosomes, which can occur simply through the shifting of a sex-specific recombination hotspot away from the sex-determining locus [34–37].

Sex chromosomes are therefore a powerful system to understand the evolutionary consequences of recombination suppression. Furthermore, detailed studies of nascent sex chromosomes are critical if we want to understand the initial causes of recombination suppression, as well as the order and rate of the evolutionary processes that follow it. For example, recent studies of young sex chromosome systems have revealed substantial intra-specific variation in the degree of recombination suppression across populations [38–41], suggesting that the boundaries of recombination suppression are not fixed within a species.

Plants in particular are useful in the study of the earliest stages of sex chromosome formation, as many plant sex chromosomes emerged only very recently in evolutionary time [42–45]. Recent studies based on next-generation sequencing of plant sex chromosomes have shown important patterns in the earliest stages of sex determination [46–51]. Studies on plant sex chromosomes have also revealed the importance of haploid selection in maintaining gene activity in the non-recombining region [17, 52] in the face of rapid loss of gene expression following recombination suppression [8, 53].

Recent work in *Salix viminalis*, the basket willow, has revealed the presence of nascent Z-W sex chromosomes, with a highly restricted SDR [54, 55]. The sex chromosomes of *Salix* have evolved independently from the X-Y system in the sister genus *Populus* [54, 56], which also exhibits very low levels of divergence between the sex chromosomes [43]. The Salicaceae family, which includes willows and poplars, therefore presents a powerful system for studying the earliest stages of sex chromosome formation. Here, we use long- and short-read single-molecule sequencing (PacBio and 10× Genomics Chromium linked-reads approaches) in *S. viminalis* to assemble a female reference genome. Importantly, our approach allowed us to obtain phased male and female haplotypes using large, continuous haplotype scaffolds. This allows us to transcend the current limitations of short-read next-generation sequencing, which hinder the assembly of repetitive regions, common in SDRs, as well as complicate accurate phasing. Our results shed unprecedented detail on the earliest stages of sex chromosome formation and reveal that the initial stages of recombination suppression are incomplete, as would be expected from gradual selection against recombination rather than from the build-up of inversions in the SDR.

## Results and discussion

### Assembly and annotation of the basket willow reference genome

In order to gain a better understanding of the evolution and genomic architecture of the recently formed sex chromosomes in *Salix viminalis*, we sequenced and assembled the complete genome of a single diploid heterogametic female (ZW) which was previously part of a large association mapping population [57]. To this end, we used a combination of long- and short-read single-molecule sequencing strategies and generated ~ 19 Gb of Pacific Biosciences (PacBio) long reads in a female and ~ 58 Gb of 10× Genomics linked-reads in the same female and a male (Additional File 1: Table S1). The full assembled genome has ~ 357 Mb of sequence spanning 2372 scaffolds above 1 kb in length, a scaffold N50 of ~ 1.3 Mb, and 92% of the genome in scaffolds longer than 50 kb. With this estimated genome size, our sequencing constitutes > 50× PacBio and > 160× 10× Genomics coverage for autosomes, and > 25× and > 40× coverage of the W chromosome accounting for the hemizygous nature of the female-limited region.

Assembly quality, as assessed by whole-genome DNA and transcriptome short-read mapping, suggests a high completeness and contiguity with ~ 98% and ~ 84% of the reads, respectively, aligned to the assembled sequence (Additional File 1: Table S2). Importantly, we obtained a high proportion of properly paired reads (Additional File 1: Table S2). An initial assessment with BUSCO also identified more than 94% of complete core Embryophyta genes in the assembly (Additional File 1: Table S2). We also mapped 1987 genotype by sequencing (GBS) [54, 58] markers in order to verify their presence and order. Consequently, our reference genome of the basket willow *S. viminalis* is essentially complete and properly assembled. Given the inherent difficulties in assembling an ancient polyploid genome such as *S. viminalis* [59], the relative completeness of our assembly reveals the benefits of incorporating single-molecule and long-read sequencing.

Annotation of the basket willow genome followed an in-house pipeline based on MAKER v3.00.0 [60] that combined transcriptome data [55, 61], reference proteins, and ab initio predictors. We identified 36,490 gene models, with 28,212 (77.3%) of them having functional annotation, and predicted 3469 ncRNA and 1139 tRNAs (Additional File 1: Table S3). Finally, we also identified several families of repetitive elements which together represent ~ 35% of the assembly. The basket willow genome is publicly available for the community through the PopGenIE Integrative Explorer (http://popgenie.org) [62].

### Delimitation of the SDR in the female assembly

Differences between male and female genomes in read depth or single nucleotide polymorphim (SNP) density can be used to identify different forms of sex chromosome divergence [12, 63]. In nascent sex chromosome systems, this method is particularly useful when combined with genetic mapping studies of sex-determining regions [41, 55]. These methods are based on the different patterns of divergence and gene coverage differences between males and females on the sex chromosomes. In female heterogametic systems, W-specific reads are present only in females, resulting in higher female read coverage for W scaffolds. Conversely, as the W degrades, we expect a greater male read depth for the corresponding region of the Z chromosome, as females retain only one copy of the Z. Additionally, in the earliest stages of recombination suppression, we expect W regions to retain significant similarity to the Z chromosome, and therefore, females may show similar read coverage for these regions as males. However, once recombination is halted, the W is expected to accumulate polymorphisms that are not shared with the Z, and so we might expect a greater density of SNPs in females compared to males in these regions even before significant divergence lowers mapping efficiency.

**Fig. 1:**
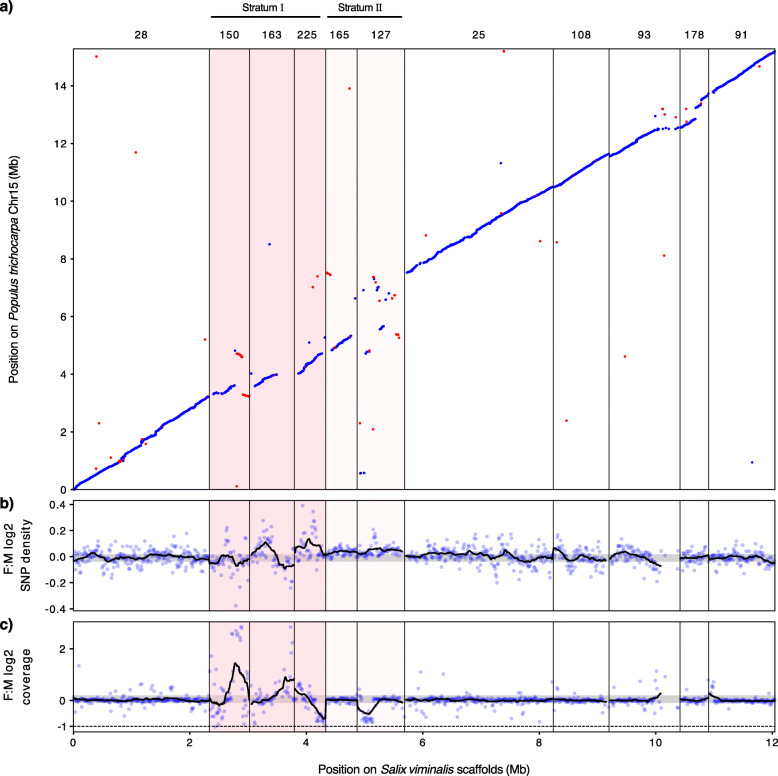
Identification of two evolutionary strata in the sex-determining region (SDR) of *S. viminalis*. Chromosome positions for *S. viminalis* and *P. trichocarpa* are shown in Mb with the *S. viminalis* scaffold names shown on the top. The two identified strata are shown with different hues of pink and labeled above the plot. **a** Anchoring of *S. viminalis* scaffolds to the autosomal chromosome 15 of *P. trichocarpa*. Forward alignments are drawn in blue and reverse alignments are drawn in red. **b** Log_2_ differences of normalized SNP density between *S. viminalis* females and males in non-overlapping windows of 10 kb. A moving average of 25 windows is shown in the black line. The gray shaded area corresponds to the bootstrap 95% confidence interval of the autosomal data. **c** Log_2_ differences of normalized read coverage between females and males in non-overlapping windows of 10 kb. Moving average and bootstrap statistics are as in **b**. Values close to −1 indicate twice the coverage in males in comparison with females, thus potentially Z-linked

In order to assess these different degrees of sex chromosome divergence, we mapped male and female short-read DNA-seq data (~ 69× and ~ 66× average sequencing coverage for females and males, respectively) to our female assembly. Because we assembled the genome of a heterogametic Z-W female, and given the relatively high levels of heterozygosity across the genome (~ 0.5% or 1 SNP per 200 bp), we expect a proportion of divergent regions in the genome, including Z and W haplotypes, to assemble separately in different scaffolds. As this would likely bias our SNP density estimates, where regions with elevated numbers of polymorphisms would appear to be homozygous, we first constructed a non-redundant assembly by removing smaller scaffolds that showed strong evidence of sequence overlap with longer scaffolds. We then aligned our non-redundant scaffolds to the *Populus trichocarpa* genome [64], revealing broad synteny as expected between these sister genera (Additional File 1: Fig. S1, Fig. 1a). In total, we anchored ~ 272 Mb (76.4% of the full assembly) to *P. trichocarpa* chromosomes.

We previously identified chromosome 15 as the sex chromosome [54, 55] and mapped the extent of the SDR on this chromosome (highlighted in pink, Fig. 1). Our results show that the five scaffolds within the SDR show significant deviations relative to the autosomal or pseudo-autosomal scaffolds of both female:male SNP density (*p* < 0.0001, one-sided *p* value from 10,000 permutations), indicative of the build-up of female-specific SNPs on the W, and/or female:male read coverage differences, suggesting regions of significant divergence between the Z and W chromosomes (Fig. 1). It is important to note that because *S. viminalis* exhibits only a limited divergence between the Z and W, and our long-read assembly was based on a female sample, the assembly of the sex chromosome regions likely represents Z-W chimeras. Chimeras such as this can obscure signals of Z-W divergence, particularly if sliding windows are very large. This chimerism is evident in scaffolds 150 and 163, which both show a region of similar coverage in males and females and a region of strong female bias that likely represents W-specific genetic material (Fig. 1). These scaffolds, in addition to scaffold 225, show the greatest deviations in the read depth between males and females and likely represent a region where recombination was first suppressed between the emerging Z and W chromosomes (stratum I). Our previous linkage mapping of the same population with GBS markers [54, 58] also placed scaffold 127 together with scaffolds 163 and 225 in the SDR (Additional File 1: Fig. S2). However, the former scaffold shows far fewer differences in female:male read depth while having higher polymorphism in females relative to males. As a result, this likely represents a region where recombination has been suppressed very recently or remains partially incomplete (stratum II).

**Fig. 2:**
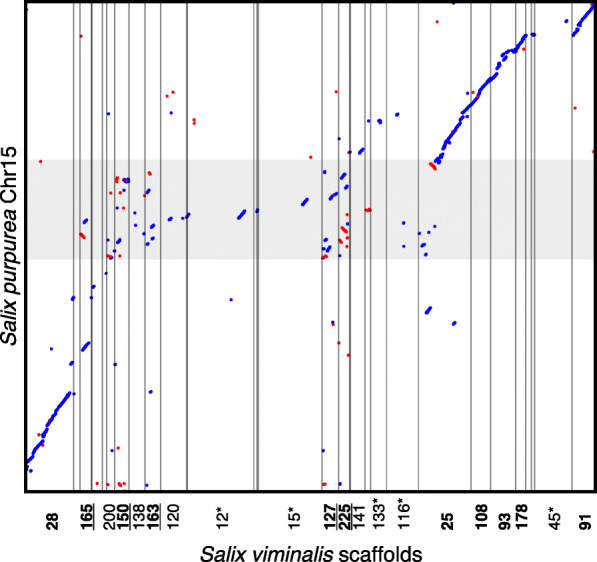
Alignment between *S. viminalis* and *S. purpurea* SDR regions. One-to-one orthologous alignments between *S. viminalis* scaffolds and chromosome 15 of *S. purpurea*, with forward alignments drawn in blue and reverse alignments drawn in red. The SDR region of *S. purpurea* is delimited by the gray shaded area (10.7–15.3 Mb, from Zhou et al. [66]). *S. viminalis* scaffolds anchored to chromosome 15 of *P. trichocarpa* are highlighted in bold, those inferred to be part of the *S. viminalis* SDR are underlined, and scaffolds well anchored with several colinear markers on other chromosomes are marked with an asterisk

The SDR region spans a total of ~ 3.4 Mb, or ~ 3.1 Mb when excluding the putatively chimeric regions, and this estimate is somewhat smaller than that of our previous estimation of ~ 5.3 Mb [55]. This difference is likely due to the fact that our previous estimate was based on a male assembly and included non-aligned regions on chromosome 15 of *P. trichocarpa*. In *Salix purpurea*, a close relative of *S. viminalis* with a divergence time of ~ 10 million years [65], the SDR is also located on chromosome 15; however, it is much larger (> 10 Mb) [66]. It has been suggested that these sex chromosomes share a common origin [66], although it remains unclear whether the SDR in these two species is in the same syntenic region. In order to test whether the SDR regions overlapped between the lineages leading to these the two species, we aligned our *S. viminalis* genome assembly to the *S. purpurea* assembly. We found that all scaffolds inferred to be part of the *S. viminalis* SDR aligned to the SDR region in *S. purpurea* (Fig. 2), suggesting a shared origin, albeit with several potential rearrangements between them.

### Two evolutionary strata on the *S. viminalis* sex chromosomes

**Fig. 3:**
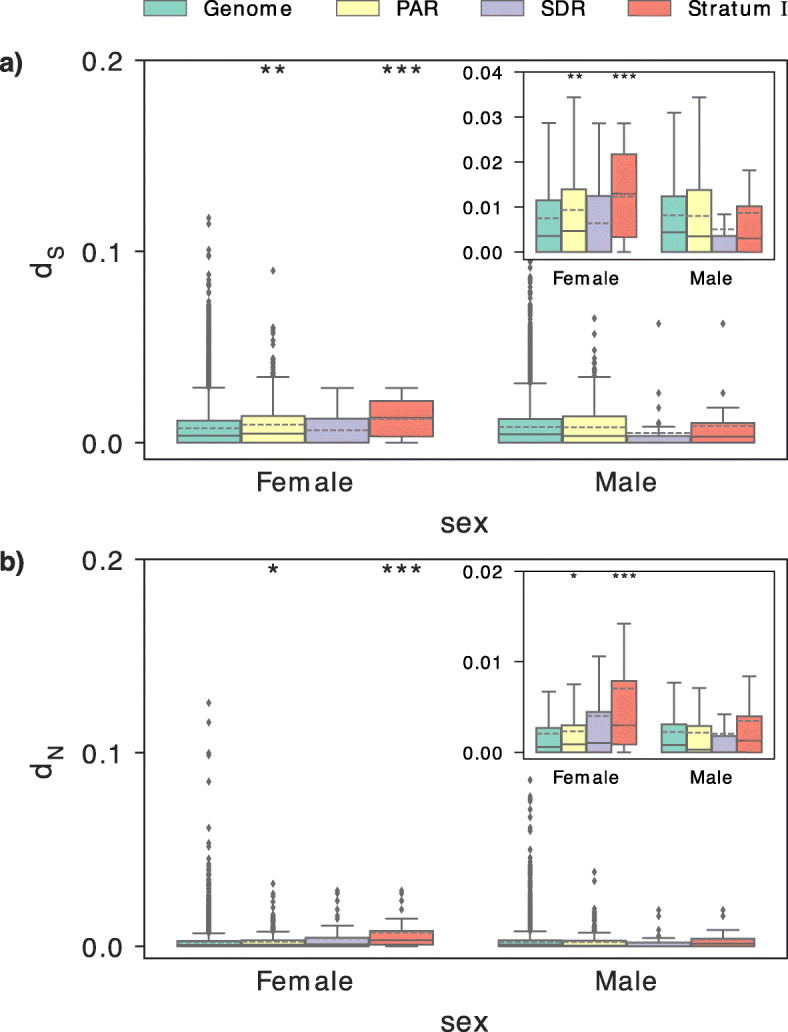
Comparison of polymorphisms at synonymous (*d*_S_) and non-synonymous (*d*_N_) sites. **a** Boxplots of d_S_ estimates. **b** Boxplots of *d*_N_ estimates. *d*_S_ and *d*_N_ were calculated based on the coding sequence alignment of phased diploid haplotypes from one female and one male individuals in the genome (excluding chromosome 15), the pseudo-autosomal region (PAR), the sex-determining region (SDR), and the more divergent stratum I. The inset plots show the quartile distributions of *d*_S_ and *d*_N_ estimates without outliers. Significant values from the Mann-Whitney *U* test relative to the genome are indicated with asterisks: *p < 0.05; **p < 0.01; ***p < 0.001

It is possible to quantify divergence between the sex chromosomes by comparing *d*_N_ (a measure of non-synonymous divergence) and *d*_S_ (a measure of synonymous divergence) between males and females in the sex-linked region. To accurately estimate this divergence, we constructed 10× Genomics Chromium de novo assemblies using one individual of each sex. Fully phased diploid genotypes were obtained for 65.8% and 61.6% of the genome in our female and the male samples, respectively. Similar phasing efficiency was also achieved for chromosome 15 (Additional File 1: Fig. S3) and for genes on the SDR, resulting in an average of 137 (63.7%) and 113 (52.5%) of genes phased in the female and male diploid assemblies, respectively. Our results show significantly greater *d*_N_ and *d*_S_ between stratum I and the genomic average in our female sample, but not in our male sample (genomic averages in female *d*_S_ 0.007460, female *d*_N_ 0.002080, male *d*_S_ 0.008151, male *d*_N_ 0.002261; stratum I female *d*_S_: mean = 0.012286, *p* = 0.00072; female *d*_N_: mean = 0.007036, *p* = 0.000077; stratum I male *d*_S_: mean = 0.008700, *p* = 0.65; male *d*_N_: mean = 0.003456, *p* = 0.25, based on Mann-Whitney *U* test relative to the genome, Fig. 3), indicating low but detectable divergence between the Z and W in this region. When stratum II is also included, the SDR shows a marginally non-significant divergence in the female (female *d*_S_: mean = 0.006402, *p* = 0.89; female *d*_N_: mean = 0.004020, *p* = 0.061; male *d*_S_: mean = 0.005052, *p* = 0.99; male *d*_N_: mean = 0.002084, *p* = 0.94, Mann-Whitney *U* test relative to the genome) despite the presence of sex-linked markers in this region (Additional File 1: Fig. S2), reinforcing the conclusion that either recombination was suppressed very recently in this region, or is not yet entirely complete. These estimates are comparatively lower than those obtained in many other plant systems [67, 68]. With the exception of the higher *d*_S_ in stratum I, they are also similar to those estimated in *S. purpurea* [66]. *d*_N_ and *d*_S_ were also marginally significantly higher between the pseudo-autosomal region (PAR) and the genome in females (*d*_S_: mean = 0.009345, *p* = 0.0019; *d*_N_: mean = 0.002308, *p* = 0.0133, Mann-Whitney *U* test), but not in males (*d*_S_: mean = 0.007991, *p* = 0.93; *d*_N_: mean = 0.002176, *p* = 0.94, Mann-Whitney *U* test).

**Fig. 4:**
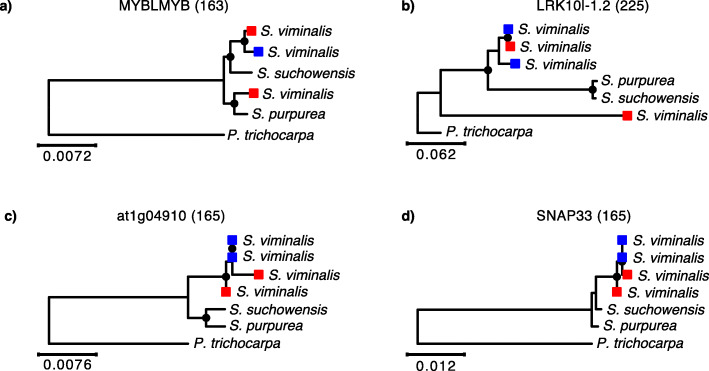
Examples of phylogenetic trees between gametologous gene pairs in the basket willow SDR. **a**, **b** The W-linked copy of the female gametolog is more divergent and does not cluster with the other *S. viminalis* haplotypes, indicating that suppression of recombination in stratum I occurred prior to *S. viminalis* speciation. **c**, **d** The female W-linked copy clusters within the species’ branch suggesting that recombination has been halted more recently. Female *S. viminalis* gametologs are indicated with red squares, and male haplotypes are in blue. Trees were estimated by maximum likelihood. Bootstrap values > 75% are indicated with black dots on the respective nodes. The poplar (*P. trichocarpa*) ortholog was used to root the trees

Phylogenetic analysis of Z-W orthologs in conjunction with outgroup species can reveal the relative timing of recombination suppression [13]. We therefore used our phased male and female haplotypes in the SDR together with orthologous genes from two closely related *Salix* species (*S. suchowensis* and *S. purpurea*) and poplar (*P. trichocarpa*). Our phylogenetic analyses provide further support for two evolutionary strata with different times since recombination suppression (Fig. 4, Additional File 1: Fig. S4). Phylogenies based on genes located in stratum I tend to show one female haplotype, corresponding to the W haplotype, clustering as an outgroup from the other three *S. viminalis* haplotypes (two male Z haplotypes and the female Z haplotype). This phylogenetic structure indicates that recombination ceased in stratum I prior to *S. viminalis* speciation. The phylogenetic structure in stratum II shows most female haplotypes clustered together with the male haplotypes, in line with more recent, or possibly partially incomplete, recombination suppression.

Distinct evolutionary strata are evident in many sex chromosome systems [9–13], and the mechanism behind recombination suppression, whether it is a large-scale inversion on the sex-limited chromosome [20] or a more gradual suppression of recombination [16–19], remains unclear. Crucially, males and females can differ substantially both in frequency and in location of recombination hotspots [69–72], referred to as heterochiasmy. Local sex-specific recombination rates within the genome may be important in both initial sex chromosome divergence and subsequent expansion of the non-recombining region [28]. Importantly, once recombination has been halted around the SDR in the heterogametic sex, selection to maintain gene order is abolished [73], and selection against inversions is greatly reduced. This suggests that inversions might follow recombination suppression, as has been recently observed [35], even if they are not the cause of recombination suppression initially.

**Fig. 5:**
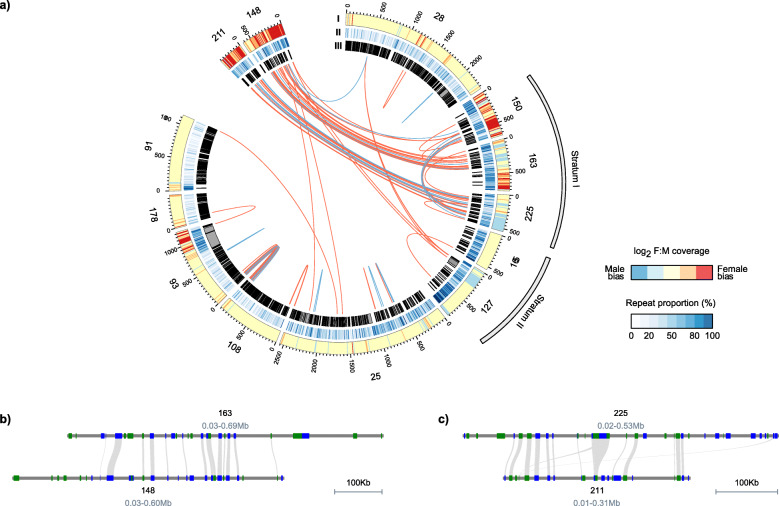
Synteny analyses of Z- and W-linked resolved haplotypes. **a** Circular plots showing that scaffolds 148 and 211 are W-linked and align to the SDR of chromosome 15. From the outside to the center, (I) depicts the heatmap of log_2_ females:males read depth in non-overlapping windows of 5 kb, (II) shows the repeat proportion in non-overlapping windows of 10 kb, and (III) indicates the location of annotated genes. Links between genes were computed from the best BLASTP hits and are color coded relative to the BLASTP alignment percent identity, with percent identity > 80% in blue and > 90% in red. Positions are shown in kb. **b**, **c** A highly conserved synteny between Z- and W-linked scaffolds

If inversions are the cause of recombination suppression between the Z and W, we would expect our female assembly to be heterozygous for inversions between the Z and W chromosomes in the SDR. To identify potential structural variations between the Z and W chromosomes, we mapped the female 10× Genomics sequencing reads to the reference genome with the barcode-aware Long Ranger pipeline from 10× Genomics. With this approach, we could detect several heterozygous deletions that largely overlap with the differences in coverage between females and males, but, consistent with the conserved synteny between the homologous Z- and W-linked scaffolds (Fig. 5, see below), we observe no evidence for inversions associated with either stratum I or stratum II (Additional File 1: Table S4).

It is still possible that inversions formed within the few remaining breakpoints in between our scaffolds, which we would not be able to detect. However, it is worth noting that we observed considerable overlap in both *d*_S_ and *d*_N_ estimates (Fig. 3) between the two strata and also the incomplete segregation of some female stratum I Z and W haplotypes (Fig. 4, Additional File 1: Fig. S4), suggesting a gradual divergence with residual or ongoing recombination in the sex chromosomes of *S. viminalis*. This gradual divergence is not consistent with a major inversion, which would result in a more similar phylogenetic signal for all Z-W orthologs within the inversion as recombination would be suppressed at the same time. Older sex chromosomes also show substantial variation in divergence within perceived strata [10, 13]; however, the limited number of loci remaining on the oldest regions of the sex-limited chromosome complicates these analyses. In these older systems, strata may also have formed through shifts in sex-specific recombination hotspots, resulting in gradual expansions rather than large-scale inversion events.

Together, our evidence suggests that at the earliest stages of sex chromosome formation and expansion, recombination suppression is a gradual process and may result from changes in sex-specific recombination hotspots or from epigenetic variation [74, 75]. Therefore, theoretical models about local changes in heterochiasmy as a result of sexually antagonistic alleles [71, 72] may prove to be key to sex chromosome evolution. Alternatively, recent evidence from fungal mating-type chromosomes, analogous to sex chromosomes in many ways, has suggested non-adaptive explanations for the origin and expansion of the non-recombining region due to neutral rearrangements [36, 76] or shifts in recombination hotspots [35].

These non-adaptive models may also explain some of the curious intra-specific heterogeneity in the extent of sex chromosome divergence in younger systems [38–41]. If recombination suppression occurs more gradually, population-level differences in sex-specific recombination hotspots, often observed [70], will drive different levels of divergence in the earliest stages of sex chromosomes, leading to inter-population differences in sex chromosome divergence.

### Degeneration of the W chromosome

Although studies of old, highly degenerate Y and W chromosomes have revealed the accumulation of significant repetitive DNA [77, 78], it remains unclear how quickly this material accumulates after recombination suppression. Additionally, the build-up of repetitive elements on the W chromosome may in itself act as a mechanism to suppress recombination with the corresponding region of the Z [79–81]. Repetitive sequences can also trigger the recruitment of the DNA methylation and histone modification machine, and by an increase in heterochromatinization indirectly promote recombination suppression in the sex chromosomes [75, 82–84]. However, the difficulty associated with phasing short-read data has previously hampered efforts to study the earliest stages of sex chromosome divergence. Although it is possible to identify sex-specific transcripts from pedigrees based on inheritance through familial pedigrees [67, 85–87], this method misses non-coding sequence, making it difficult to assess whether non-coding repetitive elements are associated with the earliest stages of recombination suppression.

In order to identify a W-specific sequence, we mapped female and male sequencing reads to our female assembly. We were able to identify an additional subset of 35 scaffolds spanning ~ 3.3 Mb and with 119 protein-coding genes (Additional File 1: Table S5), which likely represent W-specific sequence, i.e., with a significant excess of female:male read coverage over the entire scaffold length based on genomic confidence intervals. Despite the recent origin of recombination suppression, these scaffolds show a significant enrichment of repetitive sequences in comparison with both the corresponding Z-linked portion of the SDR and the genomic average (Additional File 1: Fig. S5, W genome *p* < 1 × 10^−46^; W-SDR *p* = 0.00058, Mann-Whitney *U* test). These results suggest that either repetitive sequence can accumulate very quickly following the arrest of recombination, or alternatively repetitive elements may in fact act to halt recombination in the absence of inversions.

The loss of recombination on the sex-limited SDR has important evolutionary effects, namely the build-up of deleterious variation and repetitive elements, as well as the loss of gene activity [6–8]. The latter effect in particular can lead to profound differences in gene content between X and Y or Z and W chromosomes in older sex chromosome systems [6]. Studies in other plant sex chromosomes have indicated that gene loss occurs in the SDR [8, 53], however, it remains unclear how quickly this occurs. Additionally, the extended haploid phase in plants may prevent loss of SDR genes expressed in the haploid phase [17, 52].

In order to identify gene content differences between the Z and the W chromosome, we used two of the W-linked scaffolds identified above, scaffolds 148 and 211. These scaffolds align almost entirely to the SDR where read mapping coverage is male-biased (Z-linked), as would be expected for sex-linked homologous regions (Fig. 5a). In both cases, we observed a high degree of synteny in the aligned regions, indicating that both gene content and gene order are still largely conserved between Z and W homologs, even in the most divergent region of the SDR (Fig. 5b, c). This is likely a function of both the recent divergence of this sex chromosome system [55], as well as the preservative effects of haploid selection on genes expressed in plant reproductive tissues. Nevertheless, seven protein-coding genes on the corresponding Z-linked scaffolds with known products are missing from the W assembly. Using a translated BLAST search of these proteins to the corresponding Z-linked scaffolds and considering a minimum query coverage of 80%, we inferred that at least two of them (os02g0180000 on scaffold 163 and TIR on scaffold 225) have likely been pseudogenized on the W. These results suggest that gene loss can occur very quickly, even in nascent sex chromosome systems.

### Candidate sex determination genes in *S. viminalis*

We scanned for genes unique to the *S. viminalis* W chromosome, or without preserved synteny to the Z homolog, as potential candidates for sex-determining loci in *S. viminalis*. We recovered several genes located on the W-linked scaffold 148 that could possibly be involved in this role, including WOX1, two genes in tandem of the two-component response regulator implicated in phytohormone signaling, ARR5 and ARR17, and three pre-mRNA splicing factor RNA helicases of the ESP3 gene family (Table 1). We could not recover ortholog copies of ARR17 and ESP3_2 in the genome, or evidence for a pseudogene in the Z chromosome, suggesting that they could have originated through either a translocation to the W or gene loss on the Z.

**Table 1:** Genes on W chromosome scaffolds 148 and 211 with non-preserved synteny relative to the homologous region on the Z chromosome. Orthologs were searched with BLASTP using an *e* value threshold of 1 × 10^−3^ and 75% minimum sequence identity

Scaffold	Gene	Product	Scaffold of the best ortholog (location in *P. trichocarpa*)
211	ADT2	Arogenate dehydratase/prephenate dehydratase 2, chloroplastic	100 (Chr08)
211	30220	Hypothetical protein	
211	30217	Hypothetical protein	
211	POPTR_0012s05040g	l-Ala-d/l-amino acid epimerase	
211	30210	Hypothetical protein	71 (Chr18)
211	FBA	Fructose-bisphosphate aldolase	402* (Chr15)
148	KP1_5	Kinesin KP1	150 (Chr15)
148	ESP3_4	Pre-mRNA-splicing factor ATP-dependent RNA helicase DEAH1	127 (Chr15)
148	CDC48MEE29	Cell division cycle protein 48 homolog	47 (Chr12)
148	ESP3_2	Pre-mRNA-splicing factor ATP-dependent RNA helicase DEAH1	
148	ESP3_6	Pre-mRNA-splicing factor ATP-dependent RNA helicase DEAH1	127 (Chr15)
148	ARR5_2	Two-component response regulator ARR5	25 (Chr15)
148	ARR17	Two-component response regulator ARR17	
148	WOX1_4	WUSCHEL-related homeobox 1	150 (Chr15)
148	ATM_6	Serine/threonine-protein kinase ATM	25 (Chr15)
148	BADH4_2	Betaine aldehyde dehydrogenase, chloroplastic	326 (Chr12)
148	ZDS_7	Zeta-carotene desaturase, chloroplastic/chromoplastic	593 (Chr15)
148	At4g28100	Uncharacterized GPI-anchored protein	12 (Chr12)
148	27648	Hypothetical protein	
148	CDKE-1_12	Cyclin-dependent kinase E-1	4 (Chr01)
148	27660	Hypothetical protein	

*Scaffold 402 was inferred as an allelic variation of scaffold 150

DEAD-box ATP-dependent RNA helicase homologs are known to regulate the formation of male gametophytes in rice [88] and have been recently identified in the candidate sex-determining region of grapevine [89]. WOX1 is a WUSCHEL-related homeobox protein, involved in the cytokinin regulatory pathway that coordinates stem cell proliferation with differentiation [90]. The *Silene latifolia* homolog of WOX1, SlWUS1, is also sex-linked on the X chromosome with the homologous copy in the Y chromosome likely lost [91]. ARR proteins are one of the final targets of the cytokinin signaling system, which is known to play important roles in flower development and floral sex differentiation in several plant species [92]. In particular, a C-type cytokinin response regulator that acts as a dominant suppressor of carpel development, resulting in female lethality, was specifically identified as the possible male sex-determining gene in kiwifruit [93, 94], and a regulator of the cytokinin metabolism is also a major potential candidate for sex determination in grapevine [89, 95]. Interestingly, within the Salicaceae family, sex-linked polymorphisms in the poplar genome map to a small region on chromosome 19 that includes ARR17 [43, 96]. Similarly, the sex-determining region of the purple willow, *S. purpurea*, appears to contain palindromic sequences of ARR17 that could be involved in sex determination [97].

Taken together, these observations suggest that cytokinin regulators are likely candidates for major sex-determining genes in *S. viminalis*, and it is possible that phytohormone signaling mediated by cytokinin regulators plays an important role in the sex determination cascade of this species. Additionally, it is reasonable to speculate on the evolutionary convergence in the control of genetic sex determination in plants as similar genes are likely being independently recruited in different plant families for these functions. Further functional and comparative studies will help to elucidate if these mechanisms are shared among the Salicaceae or, alternatively, evolved independently in the different groups.

It is worth noting that dioecy evolved early in the Salicaceae lineage in which *S. viminalis* is embedded and is shared by most members of the clade [98]. This means that the standard model for the evolution of sex chromosomes in plants, which assumes an immediate hermaphrodite ancestor, may not be applicable. The model posits two linked mutations encoding male and female sterility [99] as the progenitor of sex chromosomes, and this model has received some empirical support [47]. However, the ancient dioecy found in Salicaceae and the observation of small and heterogeneous levels of divergence in the basket willow [55] and poplar [43] sex chromosomes are difficult to reconcile with this two-gene model, although it is of course still possible. Indeed, recent work has pointed out alternative sex determination mechanisms in flowering plants, either determined by a single gene as in the case of *Diospyros* [46] or, as in *Cucumis* and *Mercurialis*, as a polygenic trait controlled by several genes distributed across different chromosomes [100, 101]. The Salicaceae family with its young sex chromosomes derived from ancient dioecy therefore provides a valuable comparative system to elucidate this process.

## Conclusions

Here, we use multiple types of single-molecule sequencing to assemble the genome of the basket willow, *S. viminalis*, and used this to reveal the earliest stages of sex chromosome evolution. This approach allows us unprecedented power to phase our data, allowing us to resolve Z and W haplotypes at this early stage of divergence. Our results suggest that the SDR is of limited size and divergence, and we recover no evidence that recombination suppression is due to inversions in this region. Even at this early stage of divergence, we see evidence of pseudogenization and the accumulation of repetitive elements in the SDR, suggesting that these processes occur very swiftly after recombination ceases. Furthermore, we found W-linked genes involved in cytokinin regulation, suggesting that phytohormone signaling could be important in the sex determination cascade in *S. viminalis*. In total, our results shed new light on the fundamental process of sex chromosome formation.

## Methods

### Plant material and DNA extraction

Fresh young leaves (approximately 200 mg) were sampled from a female and a male *S. viminalis* (accession 78183 and 81084, respectively), described in [57, 102], and DNA was extracted following a CTAB protocol described in [55]. In brief, approximately 200 mg fresh leaves were snap-frozen and pulverized. To every sample, 950 μl of extraction buffer (100 mM Tris-HCl pH 7.5–8, 25 mM EDTA, 2 M NaCl, 2% (w/v) CTAB, 2% (w/v) PVP K30, 5% (w/v) PVPP, 50 μg/ml RNAse) was added, and the sample was thoroughly mixed before incubation for 30 min at 65 °C. Subsequently, 300 μl chloroform to isoamyl alcohol 24:1 was added, the sample mixed and centrifuged for 10 min at 13,000 rpm, the supernatant was transferred to a new tube, and the process was repeated. 1.5 volumes of ice-cold isopropanol were added to the supernatant followed by an incubation overnight at − 20 °C. After centrifugation for 10 min at 13,000 rpm at 4 °C, the supernatant was removed and the pellet rinsed with chilled 100% EtOH followed by another centrifugation of 5 min at 13,000 rpm at 4 °C. The supernatant was then removed and the DNA was air-dried before it was dissolved in 100 μl TE buffer (10 mM Tris-HCl, 1 mM EDTA). DNA concentration was assessed by Qubit 3.0 Fluorometer (Thermo Fisher Scientific).

### PacBio long-read library preparation and sequencing

A single SMRT-bell library with 20 kb insert size was constructed from 10 μg of pure high-molecular weight DNA from one *S. viminalis* female (accession 78183) according to the manufacturer’s protocol (Pacific Biosciences). This library was sequenced on 48 SMRT cells using P5-C3 chemistry, and 4-h movies were captured for each SMRT cell using the PacBio RSII sequencing platform (Pacific Biosciences). Primary analysis and error correction of the raw data were done using SMRT Portal (Pacific Biosciences). After filtering, the mean read length was 8924 bp (longest read was 61 kbp) and a total of ~ 19.2 Gbp of data were recovered.

### 10× Genomics Chromium linked-reads library preparation and sequencing

For both accessions (78183 and 81084), sequencing libraries were prepared from 0.75 ng DNA using the Chromium TM Genome Library preparation kit according to the CG00022_Chromium Genome Reagent Kit User Guide_RevA. The library preparation was performed according to the manufacturers’ instructions with the exception that 0.75 ng was used for library preparation instead of 1.25 ng recommended by the manufacturer’s instructions. This was done to account for the smaller genome size of *S. viminalis* compared to the human genome for which the protocol was optimized. The libraries were sequenced on an Illumina HiSeqX with a paired-end 150-bp read length using v2.5 sequencing chemistry (Illumina Inc.), resulting in ~ 58 Gb of data with a mean molecule length of ~ 40 kb.

### DNA extraction and short-read Illumina sequencing

We generated additional Illumina sequencing data for the female accession 78183, the same accession used to assemble the reference genome. DNA was extracted from fresh leaves using the Fast DNA Kit (MP Biomedicals) according to the manufacturer’s instructions. Two libraries with 165 and 400 bp insert size respectively were generated with the TruSeq DNA v2 kit (manual #15005180) following the manufacturer’s protocol and sequenced on one lane each with Illumina HiSeq2000, 100- bp paired-end read length, and v3 chemistry generating ~ 28 Gb of bases (Additional File 1: Table S1).

### Reference genome assembly and annotation

Falcon v0.4.2 [103] was used to assemble the sub-reads from 48 SMRT cells. The primary contigs from this first draft assembly were then polished using Quiver from the Pacific Biosciences’ SMRT suite (v2.3.0) with the PacBio reads. The resulting assembly was then corrected with Pilon v 1.17 [104] using both Illumina libraries from the same individual at 80× and 53× coverage. In addition, a 10× Genomics assembly for the same female individual was also obtained using the pseudohap-style output of Supernova v2.0.1 [105]. This 10× Genomics assembly and the PacBio assembly were then merged using Quickmerge v20160905 [106], increasing the assembly size by ~ 8 Mb. Finally, the preads (corrected PacBio reads obtained after the first step of Falcon assembly) and the Supernova pseudohap assembly were used to scaffold the merged assembly using LINKS v1.8.4 [107]. Finally, we corrected some homozygous SNPs and small insertions and deletions in the assembly using Long Ranger v2.1.2 with the 10× Genomics Chromium reads of the same female individual.

Annotation of the *S. viminalis* reference genome was performed with MAKER v3.00.0 [60]. The MAKER pipeline was run twice; first, based on protein and RNA sequence data only (later used to train ab initio software) and a second time combining evidence data and ab initio predictions. High-confidence protein sequences were collected from the Uniprot database [108], for proteins belonging to the Swissprot section that contain only manually annotated and reviewed curations (downloaded on August 2016), and two other specific protein sets from *Salix suchowensis* and *Populus trichocarpa*. Furthermore, to support gene predictions, we also used selected libraries of RNA-seq data from our previous studies collected from vegetative (leaf) and sex-specific reproductive tissue (catkin) from both female and male individuals [55, 61]. As the basis for the construction of gene models, we combined ab initio predictions from three sources (Augustus v2.7 [109], GeneMark_ES_ET v4.3 [110], and SNAP [111]). GeneMark_ES_ET was self-trained with the genome sequence. To train Augustus and SNAP, we first ran the MAKER pipeline the first time to create a profile using the protein evidence along with RNA-seq data. Both Augustus and SNAP were then trained with a selected set of genes from this initial evidence-based annotation. We excluded genes with an Annotation Edit Distance (AED) score equal to 1 to avoid potentially false annotations. Functional inference for genes and transcripts was performed using the translated CDS features of each coding transcript. Protein sequences were searched with BLAST in the Uniprot/Swissprot reference dataset in order to retrieve gene names and protein functions as well as in the InterProscan v5.7-48 database to retrieve additional annotations from different sources.

We created a repeat library with an in-house pipeline using RepeatModeler v1.0.8 [112]. Identification of repeat sequences in the genome was performed using RepeatMasker v4.0.3 [113] and RepeatRunner [114]. tRNAs were predicted with tRNAscan v1.3.1 [115], and broadly conserved ncRNAs were predicted with the Infernal package [116] using the RNA family database Rfam v11 [117]. The genome assembly and annotation were converted in EMBL format using EMBLmyGFF3 [118].

### Identification of allelic scaffolds in single-molecule de novo assemblies

The linked-reads technology of 10× Genomics uses a large number of barcoded microdroplets (GEMs) to capture long-range information over long, single-DNA molecules, enabling the assembly through repetitive regions and to resolve heterozygous haplotypes from a diploid genome. Linked reads for the female and male accessions were assembled with Supernova v2.0.1 [105]. Fully phased heterozygous haplotypes, together with non-phased sequence (nominally homozygous), were obtained using the megabubbles-style output and a minimum sequence length of 1 kb. With this output style, Supernova generates an individual FASTA record for each homologous phased haplotype without mixing maternal and paternal alleles in the same sequence. Diploid assemblies were soft-masked with RepeatMasker v4.0.7 [113] with the “RMBlast” v2.6.0+ search engine and using our custom *S. viminalis* repeat library generated during genome annotation.

We used sequence alignments in order to identify homologous haplotypes in our single-molecule assemblies. A repeat-masked assembly is first aligned to itself with LAST v926 [119] using the sensitive DNA seeding MAM4 [120] and masking of repeats during alignment with the -cR11 option. To avoid false matches caused by repetitive sequences and paralogous scaffolds, orthologous alignments were generated with last-split and alignments mostly comprised of masked sequence were then discarded with last-postmask. Scaffolds were considered to represent allelic variants in the assembly if the overlap exceeded 25% of sequence length after repeat masking, and with sequence identity > 80% to other longer scaffolds.

### Anchoring scaffolds to *Populus trichocarpa*

Pairwise alignments between *P. trichocarpa* v10.1 (downloaded from PopGenie v3 [62]) and our *S. viminalis* assembly were generated from repeat-masked genomic sequence using LAST v926 [119]. We first prepared an index of the poplar genome using the sensitive DNA seeding MAM4 [120], using the masking repeat option -cR11 during alignment. A suitable substitution and gap frequencies matrix were then determined with last-train, using parameters --revsym --matsym --gapsym -C2. Alignments were made with lastal, using the parameters -m100 -C2 followed by last-split –m1 to find 1-to-many willow-poplar orthologous matches. Finally, alignments (within scaffolds) that were composed primarily of masked sequence were ignored using last-postmask, and scaffolds with less than an overall of 10% of aligned sequence were discarded. One-to-one willow-poplar alignments were made by swapping both sequences and repeating the orthology search as above.

Neighboring alignments with < 10 kb gap lengths were linked into a single path, and the longest tiling path was used to assign scaffolds to poplar chromosomes. Forward or reverse scaffold orientation relative to poplar chromosomes was similarly obtained requiring that the total length of one alignment direction was > 70% compared to the other orientation; otherwise, the original orientation was kept. If the longest tiling path for a particular scaffold did not agree with its overall alignment path on the poplar chromosome, the scaffold was marked as unlocalized.

### Preprocessing of Illumina reads

Whole-genome DNA sequencing reads were quality assessed with FastQC v0.11.5 [121] and preprocessed with BBTools v37.02 “bbduk” [122] to remove adapter sequences, to trim regions with average quality scores below Q10 from both ends of reads, and to filter out reads aligning to PhiX-174 genome (a commonly used spike-in control in Illumina sequencing runs). After filtering, read-pairs were excluded from downstream analyses if either read had an average quality score < Q20 or was < 50 bases in length. The same criteria for quality assessment and filtering were used for RNA-seq data.

### Coverage and polymorphism analysis

Alignments to the genome assembly were performed with BWA v0.7.15-r1140 using the MEM [123] algorithm and default options. General processing of SAM/BAM files was performed with SAMtools v1.6 [124], and duplicated reads were flagged with biobambam v2.0.72 [125] after alignment. Per-site coverage was computed with the SAMtools depth command after filtering out reads with mapping quality ≥ Q3 that map to multiple locations, reads with secondary alignments, and duplicated reads. We then calculated the effective coverage value per scaffold and in non-overlapping windows of 10 kb, as the mean per-site coverage of every site in that class. To account for the differences in the overall coverage between individuals, the coverage data were normalized for the median coverage value of each individual in the respective class.

Polymorphism analyses were conducted using the same filters as above. Read alignments were then converted to nucleotide profiles with the sam2pro program of mlRho [126]. Only sites with a per-site coverage ≥ 5 and a SNP called for bi-allelic sites with a minor allele frequency ≥ 30% within an individual were analyzed. The average SNP density per scaffold, and window, was calculated as the number of SNPs divided by the number of sites that passed the coverage threshold of 5 for the respective class.

In order to avoid infinitely high numbers associated with log_2_ 0 when calculating the log_2_ difference of coverage or SNP density between females and males, we added a small number (0.1) to each value. The 95% confidence intervals for the sliding window distributions were estimated from the mean bootstrap values with resampling of 1000 random sets of 25 windows from autosomes. We excluded the entirety of chromosome 15 (the sex chromosome), including the PAR, in the bootstrapping procedure to avoid potential linkage effects resulting from the SDR.

To identify potentially W-linked scaffolds in the assembly, we proceeded as above and calculated the log_2_ F:M coverage differences for each scaffold. All scaffolds where the normalized female coverage was < 10% of the normalized whole-genome coverage were excluded. This is a conservative approach because of the difficulty associated with mapping to highly repetitive potential W-linked scaffolds. These scaffolds are therefore likely to remain undetected. Scaffolds were considered W-linked if the log_2_ F:M coverage difference was > 95% the genome average.

### Identification of structural variations

We used the linked-read data of 10× Genomics sequencing to search for large structural variations in the sex-determining region of the female assembly, as long-range information (average molecule length of ~ 40 kb) can provide accurate sequencing information spanning the region around a breakpoint, even if breakpoints are in regions that are inherently difficult to assemble, for example, in areas enriched for repetitive sequence. To facilitate the interpretation of structural variants that could span adjacent scaffolds, we concatenated all scaffolds anchored to chromosome 15 into a single pseudo-chromosome, preserving scaffold ordering, separated by runs of 100 null nucleotides (N). We then used Long Ranger (10× Genomics), which employs barcode-aware read alignments for the identification of large-scale structural variations using the 10× linked-reads library of the same female used in the assembly (accession 78183). Read mapping used the full genome assembly; however, structural variants were called specifically on the pseudo-chromosome 15.

### Quantification of gene expression

Preprocessed RNA-seq reads [55, 61] were filtered for rRNA using Bowtie v2.3.2 [127], and the SILVA release 128 database of LSU and SSU NR99 rRNAs [128]. Filtered reads were then aligned to the reference assembly using HISAT2 v2.1.0 [129] with options --no-mixed --no-discordant. The resulting alignments for each library were sorted and merged by individual and by tissue (catkin and leaves) with SAMtools v1.6 [124]. Read counting per gene was performed using the count command of HTSeq [130] and reads per kilobase mapped (rpkm) expression values were calculated with edgeR [131]. Only genes with an rpkm 1 in at least one sample were considered in further analyses.

### Annotation lift-over to 10× Genomics diploid assemblies

Our reference genome annotation was transferred independently for each of the inferred haplotypes derived from our 10× Genomics de novo assemblies of female and male genomes using UCSC Genome Browser’s utilities [132]. First, a pairwise alignment between each haplotype and the non-redundant reference genome was generated as described above with LAST v926 [119]. Alignments were then converted into a series of syntenic chains and nets, tuned for more divergent genomes (axtChain -linearGap=loose), using the same scoring matrix generated during the LAST alignments. Finally, annotations were moved to the haplotype assemblies using the liftOver utility with a minimum 75% ratio of mapped bases between features. Only the longest isoform of each gene was considered in the lift-over. With this approach, we transferred ~ 25,159 genes per diploid haplotype or ~ 80% of the complete annotation.

We further attempted to recover additional genes not lifted initially by aligning each gene individually back to the haplotype assemblies with BLAT v170523 [133], (-minIdentity=30 -minScore=12 -stepSize=5 -repMatch=2253 -extendThroughN), keeping the highest-scoring alignment for each query. In order to avoid potential problems caused by the BLAT alignment of paralogous sequences, we counted the average number of haplotypes aligned to each reference gene (for a fully phased diploid region we expect 2 haplotypes). These counts were then bootstrapped with 1000 iterations, and all alignments for which the haplotype coverage was below the lower bootstrap 95% confidence interval (~ 1.6× coverage) were excluded. This procedure recovered an average of 364 additional genes per haplotype.

### Divergence analysis of diploid genotypes

We calculated rates of divergence at synonymous (*d*_S_) and non-synonymous (*d*_N_) sites between the coding sequences of diploid genotypes for each sex separately. In order to further increase the number of genes analyzed in the SDR, we additionally made use of the resolved haplotypes derived from the female PacBio assembly (from scaffolds 163 and 225). We identified orthologous genes with BLASTP using an *e* value threshold of 1 × 10^−3^ and only considering the top hit for each gene. This process recovered an additional set of 11 genes that have not been previously lifted-up to the 10× Genomics diploid assembly. Only sequences with a valid start codon, without internal stop codons and with a minimum sequence length of 120 bases, were analyzed. After this initial filter, pairwise alignments for the two haplotypes were obtained with PRANK v140603 [134], and *d*_S_ and *d*_N_ estimates were calculated using the method of Yang and Nielsen [135] as implemented in the yn00 program of PAML v4.9h [136]. Pairwise comparisons with *d*_S_ > 0.2 were excluded, thereby avoiding the incorrect assignment of orthologs.

### Phylogenetic analysis

We used gene trees to determine the relative age of recombination suppression for the haplotypes in each identified sex chromosome strata. In addition to our non-redundant *S. viminalis* genome, coding sequences for *S. suchowensis* v4.1 and *P. trichocarpa* v10.1 were obtained from PopGenie v3 [62] and sequences for *S. purpurea* v1.0 were obtained from Phytozome v12 [137]. Only the longest transcripts were considered. We first use the conditional reciprocal best BLAST method [138], with a BLAST *e* value cutoff < 1 × 10^−5^, to identify 14,255 one-to-one orthologs across all four species (*S. viminalis*, *S. suchawensis*, *S. purpurea*, and *P. trichocarpa*). For each ortholog group, we searched for the *S. viminalis* homolog in the lifted annotation of the female and male phased diploid assemblies and aligned all species’ sequences with MAFFT v7.313 [139]. Aligned columns with > 40% gaps and taxa with > 40% of missing data were removed. Maximum likelihood phylogenetic trees were obtained with RAxML v8.2.12 [140] using the rapid bootstrap algorithm with 100 bootstraps and the GTRGAMMA model of sequence evolution. Trees were rooted in the *P. trichocarpa* branch and were only considered if the two female haplotypes were present. Phylogenetic tree analyses were performed with ETE3 [141].

## Supplementary information

**Additional file 1: Table S1.** Whole-genome DNA sequencing data used in this study. **Table S2.** Assembly statistics for the full and non-redundant assemblies of *Salix viminalis*. **Table S3.** Characterization of the different annotation classes for the *Salix viminalis* assembly. **Table S4.** Characterization of heterozygous structural variations identified in Chr15. **Table S5.** List of all genes found on putatively W-linked scaffolds. **Figure S1.** Whole genome synteny between *Salix viminalis* assembly and *Populus trichocarpa*. **Figure S2**. Genetic markers aligned to chromosome 15 (from Pucholt et al. 2015) on our assembly. **Figure S3.** Percentage of fully phased haplotypes using 10X Genomic Chromium sequence data. **Figure S4**. Phylogenetic trees between Z-W gene pairs in the basket willow SDR. **Figure S5**. Density of repetitive elements across different genomic regions.

## Data Availability

Genome sequencing data and annotation generated for this study have all been deposited in EBI’s ENA (https://www.ebi.ac.uk/ena) under project number PRJEB31619 [142]. The datasets used and/or analyzed during the current study are available from the corresponding author on reasonable request.
