# Supplementary material for: Genome assembly of the basket willow, Salix viminalis, reveals earliest stages of sex chromosome expansion
Source: BMC Biol. 2020 Jun 30;18:78. doi: 10.1186/s12915-020-00808-1 (PMC7329446; doi:10.1186/s12915-020-00808-1)

Supplementary Material

Table S1. Whole-genome DNA sequencing data used in this study.

Accession	Sex	Technology	Number of reads (millions)	Number of Megabases (Gb)	Reference
78183	Female	Pacific Biosciences	2.7	19	This study
		10x Genomics	390	59	This study
		Illumina – 165 bp insert	191	19	This study
		Illumina – 400 bp insert	289	28	This study
78021	Female	Illumina	204	26	Pucholt et al. 2017
78195	Female	Illumina	199	20	Pucholt et al. 2017
81084	Male	10x Genomics	429	64	This study
		Illumina	210	21	Pucholt et al. 2017
T76	Male	Illumina	214	27	Pucholt et al. 2017

Table S2. Assembly statistics for the full and non-redundant assemblies of *Salix viminalis*. DNA- and RNA-seq mapping were performed with read data from the same individual used in the assembly. "N" and "L" statistics were calculated at the scaffold level.

Metric	Full assembly		Non-redundant assembly	
Number of scaffolds	2,372		1,467	
Total bases (bp)	357,061,245		303,195,481	
Longest sequence (bp)	7,291,771		7,291,771	
N50	73		54	
L50 (bp)	1,314,944		1,658,965	
N90	434		203	
L90 (bp)	77,063		300,484	
% gaps (N)	0.73		0.48	
BUSCO - genome	79.5% single copy		82.3% single copy	
	15.0% duplicated		10.0% duplicated	
BUSCO - proteins	73.1% single copy		75.2% single copy	
	14.1% duplicated		9.7% duplicated	
DNA-seq mapping	98.41% (mapped)	90.45% (properly paired)	97.72% (mapped)	89.31% (properly paired)
RNA-seq mapping (2 tissues)	84.28% (mapped)	71.14% (properly paired)	81.85% (mapped)	69.16% (properly paired)

Table S3. Characterization of the different annotation classes for the *Salix viminalis* assembly.

Source	Value
Gene number	36,490
Gene span	173,927,686
Gene average length (stdev)	4,766 (10,540)
Genes with functional annotation (%)	28,212 (77.3%)
Transcript number	49,131
Transcript average length (stdev)	6,485 (16,044)
Intron number	294,271
Intron average length (stdev)	786 (4,636)
3’ UTR number	26,926
3’ UTR average length (stdev)	404 (423)
5’ UTR number	26,883
5’ UTR average length (stdev)	245 (307)
Exon number	343,402
Exon average length (stdev)	254 (341)
CDS number	49,131
CDS average length (stdev)	298 (403)
Genes with multiple transcripts (%)	6,158 (16.9%)
Average number of transcripts per gene	1.35
Average number of exons per transcript	6.99
Single exon transcripts (%)	4,367 (8.9%)
Gene coverage	48.7%
CDS coverage	4.1%
ncRNA	3,469
tRNA	1,139

Table S4. Characterization of heterozygous structural variations identified in Chr15. Structural variants were called with the 10x Genomics Long Ranger pipeline. Scaffolds anchored to chromosome 15 were concatenated into a single pseudo-chromosome with 100 N’s in between.

Chromosome	Start position	End position	Size	Type
Chr15	2,019,348	2,054,847	35,499	Deletion
Chr15	2,710,000	3,190,001	480,001	Deletion
Chr15	3,597,312	3,644,447	47,135	Duplication
Chr15	3,703,990	3,765,220	61,230	Unknown
Chr15	4,830,000	5,150,001	320,001	Deletion
Chr15	5,965,532	6,013,673	48,141	Unknown
Chr15	8,226,359	8,259,916	33,557	Deletion
Chr15	10,040,000	10,420,001	380,001	Deletion
Chr15	10,385,195	10,439,326	54,131	Deletion

Table S5. List of all genes found on putatively W-linked scaffolds. Orthologs were searched with BLASTP using an e-value threshold of 1x10-3 and 75% minimum sequence identity.

Scaffold	Gene	Product	Scaffold of best ortholog (location in *P. trichocarpa*)
211	CCT4_2	T-complex protein 1 subunit delta	225 (Chr15)
211	GRDP1	Glycine-rich domain-containing protein 1	225 (Chr15)
211	ADT2	Arogenate dehydratase/prephenate dehydratase 2, chloroplastic	100 (Chr08)
211	30233	hypothetical protein	
211	AT1g18030_2	Probable protein phosphatase 2C 8	225 (Chr15)
211	AT1g22950_3	Uncharacterized PKHD-type hydroxylase At1g22950	225 (Chr15)
211	PAPS1_2	Nuclear poly(A) polymerase 1	225 (Chr15)
211	30222	hypothetical protein	225 (Chr15)
211	GGR_2	Heterodimeric geranylgeranyl pyrophosphate synthase small subunit, chloroplastic	225 (Chr15)
211	30220	hypothetical protein	
211	30219	hypothetical protein	225 (Chr15)
211	HIP1	Probable E3 ubiquitin-protein ligase HIP1	
211	30217	hypothetical protein	
211	30215	hypothetical protein	
211	RTNLB9	Reticulon-like protein B9	225 (Chr15)
211	POPTR_0012s05040g	L-Ala-D/L-amino acid epimerase	
211	NACK1_2	Kinesin-like protein NACK1	225 (Chr15)
211	30210	hypothetical protein	71 (Chr18)
211	30206	hypothetical protein	225 (Chr15)
211	AMSH1_2	AMSH-like ubiquitin thioesterase 1	225 (Chr15)
211	30204	hypothetical protein	225 (Chr15)
211	FBA	Fructose-bisphosphate aldolase	402 (Chr15)
148	KP1_5	Kinesin KP1	150 (Chr15)
148	ESP3_4	Pre-mRNA-splicing factor ATP-dependent RNA helicase DEAH1	127 (Chr15)
148	CDC48MEE29	Cell division cycle protein 48 homolog	47 (Chr12)
148	ESP3_2	Pre-mRNA-splicing factor ATP-dependent RNA helicase DEAH1	
148	ESP3_6	Pre-mRNA-splicing factor ATP-dependent RNA helicase DEAH1	127 (Chr15)
148	ARR5_2	Two-component response regulator ARR5	25 (Chr15)

148	ARR17	Two-component response regulator ARR17	
148	WOX1_4	WUSCHEL-related homeobox 1	150 (Chr15)
148	CML27_4	Probable calcium-binding protein CML27	163 (Chr15)
148	GC2	Golgin candidate 2	163 (Chr15)
148	ATM_6	Serine/threonine-protein kinase ATM	25 (Chr15)
148	BADH4_2	Betaine aldehyde dehydrogenase, chloroplastic	326 (Chr12)
148	ZDS_7	Zeta-carotene desaturase, chloroplastic/chromoplastic	593 (Chr15)
148	HMGIY2_2	HMG-Y-related protein A	163 (Chr15)
148	MPK15	Mitogen-activated protein kinase 15	163 (Chr15)
148	AT4g28100_3	Uncharacterized GPI-anchored protein At4g28100	12 (Chr12)
148	27648	hypothetical protein	
148	MYB6	Transcription repressor MYB6	163 (Chr15)
148	CDKE-1_12	Cyclin-dependent kinase E-1	4 (Chr01)
148	AIP1-2	Actin-interacting protein 1-2	163 (Chr15)
148	BGLU44_3	Beta-glucosidase 44	163 (Chr15)
148	EXO1_2	Exonuclease 1	163 (Chr15)
148	MYBL	Myb-like protein L	163 (Chr15)
148	GB1_3	Guanine nucleotide-binding protein subunit beta-like protein	163 (Chr15)
148	PEX3_2	Peroxisome biogenesis protein 3-2	163 (Chr15)
148	MOS4_2	Pre-mRNA-splicing factor SPF27 homolog	163 (Chr15)
148	27660	hypothetical protein	
148	GSTU4	Glutathione S-transferase U4	
148	TAS_3	Protein tas	
200	29900	hypothetical protein	200
200	29899	hypothetical protein	14 (Chr06)
200	SCD1_6	DENN domain and WD repeat-containing protein SCD1	355
200	29897	hypothetical protein	127 (Chr15)
200	SCD1_6	DENN domain and WD repeat-containing protein SCD1	355
200	29895	hypothetical protein	
200	ATK4_3	Kinesin-4	
200	KP1_4	Kinesin KP1	150 (Chr15)
200	ESP3_8	Pre-mRNA-splicing factor ATP-dependent RNA helicase DEAH1	127 (Chr15)
200	ESP3_9	Pre-mRNA-splicing factor ATP-dependent RNA helicase DEAH1	28 (Chr15)

200	ARP4_3	Actin-related protein 4	
200	ARP4_4	Actin-related protein 4	127 (Chr15)
200	Y39A1A	Ribosomal RNA small subunit methyltransferase nep-1	8 (Chr16)
200	29883	hypothetical protein	165 (Chr15)
305	PDF2	Defensin-like protein 1	120
305	PDF2	Defensin-like protein 6	120
305	N_98	TMV resistance protein N	265 (Chr18)
305	N_84	TMV resistance protein N	171
305	PDR2_6	Probable manganese-transporting ATPase PDR2	384
305	N_19	TMV resistance protein N	1073
322	UBP12_7	Ubiquitin carboxyl-terminal hydrolase 12	49 (Chr17)
322	DLO2_6	Protein DMR6-LIKE OXYGENASE 2	198
322	AT5g09450_5	Pentatricopeptide repeat-containing protein At5g09450, mitochondrial	252 (Chr17)
322	AT5g09450_3	Pentatricopeptide repeat-containing protein At5g09450, mitochondrial	49 (Chr17)
322	DLO2_4	Protein DMR6-LIKE OXYGENASE 2	1173
336	AT1g64760_3	Glucan endo-1,3-beta-glucosidase 8	
343	UBP12_3	Ubiquitin carboxyl-terminal hydrolase 12	322
343	DLO2AT5g09450	Protein DMR6-LIKE OXYGENASE 2	
384	33334	hypothetical protein	171
384	N54	TMV resistance protein N	
384	PDR2MYB308	Probable manganese-transporting ATPase PDR2	319
384	AT4g27220_44	Probable disease resistance protein At4g27220	120
387	MYB330	Myb-related protein 330	
388	RGA4_58	Putative disease resistance protein RGA4	232 (Chr17)
388	BMS1_10	Ribosome biogenesis protein BMS1 homolog	232 (Chr17)
388	RGA3_3	Putative disease resistance protein RGA3	232 (Chr17)
388	RGA4_14	Putative disease resistance protein RGA4	232 (Chr17)
388	33367	hypothetical protein	
388	BMS1_5	Ribosome biogenesis protein BMS1 homolog	388
464	ACD6_15	Protein ACCELERATED CELL DEATH 6	35 (Chr13)
464	33929	hypothetical protein	35 (Chr13)
493	34069	hypothetical protein	
502	ZFN1_2	Zinc finger CCCH domain-containing protein ZFN-like	167 (Chr13)

502	PIN5_3	Auxin efflux carrier component 5	167 (Chr13)
502	CBSX6_2	CBS domain-containing protein CBSX6	167 (Chr13)
539	AT2g19130b120	G-type lectin S-receptor-like serine/threonine-protein kinase B120	17 (Chr05)
590	M3KE1_10	MAP3K epsilon protein kinase 1	26 (Chr07)
590	M3KE1_36	MAP3K epsilon protein kinase 1	79 (Chr18)
590	34462	hypothetical protein	21 (Chr02)
590	CTSB_2	Cathepsin B	21 (Chr02)
1389	35922	hypothetical protein	6 (Chr06)
464	ACD6_15	Protein ACCELERATED CELL DEATH 6	35 (Chr13)
464	33929	hypothetical protein	35 (Chr13)
563	BGAL9	Beta-galactosidase 9	65 (Chr17)
563	BGAL9NEK6	Beta-galactosidase 9	65 (Chr17)
574	AT1g07650_8	Probable LRR receptor-like serine/threonine-protein kinase At1g07650	291 (Chr01)
580	34425	hypothetical protein	580 (Chr08)
580	34427	hypothetical protein	58 (Chr08)
580	RNP1_12	Heterogeneous nuclear ribonucleoprotein 1	58 (Chr08)
595	AT3g47200_66	UPF0481 protein At3g47200	107 (Chr01)
595	OS08g0118900	Probable adenylate kinase 7, mitochondrial	107 (Chr01)
595	34484	hypothetical protein	107 (Chr01)
595	AT3g03770_5	Probable inactive leucine-rich repeat receptor-like protein kinase At3g03770	107 (Chr01)
609	WAP_2	WPP domain-associated protein	181 (Chr01)
609	FKBP16-1_2	Peptidyl-prolyl cis-trans isomerase FKBP16-1, chloroplastic	181 (Chr01)
709	XPO1	Protein EXPORTIN 1A	532
769	NFYB8_3	Nuclear transcription factor Y subunit B-8	83 (Chr16)
769	34929	hypothetical protein	77 (Chr05)

Supplemental Figure S1. Whole genome synteny between *Salix viminalis* assembly and *Populus trichocarpa*. Forward alignments are drawn in blue and reverse alignments are drawn in red.

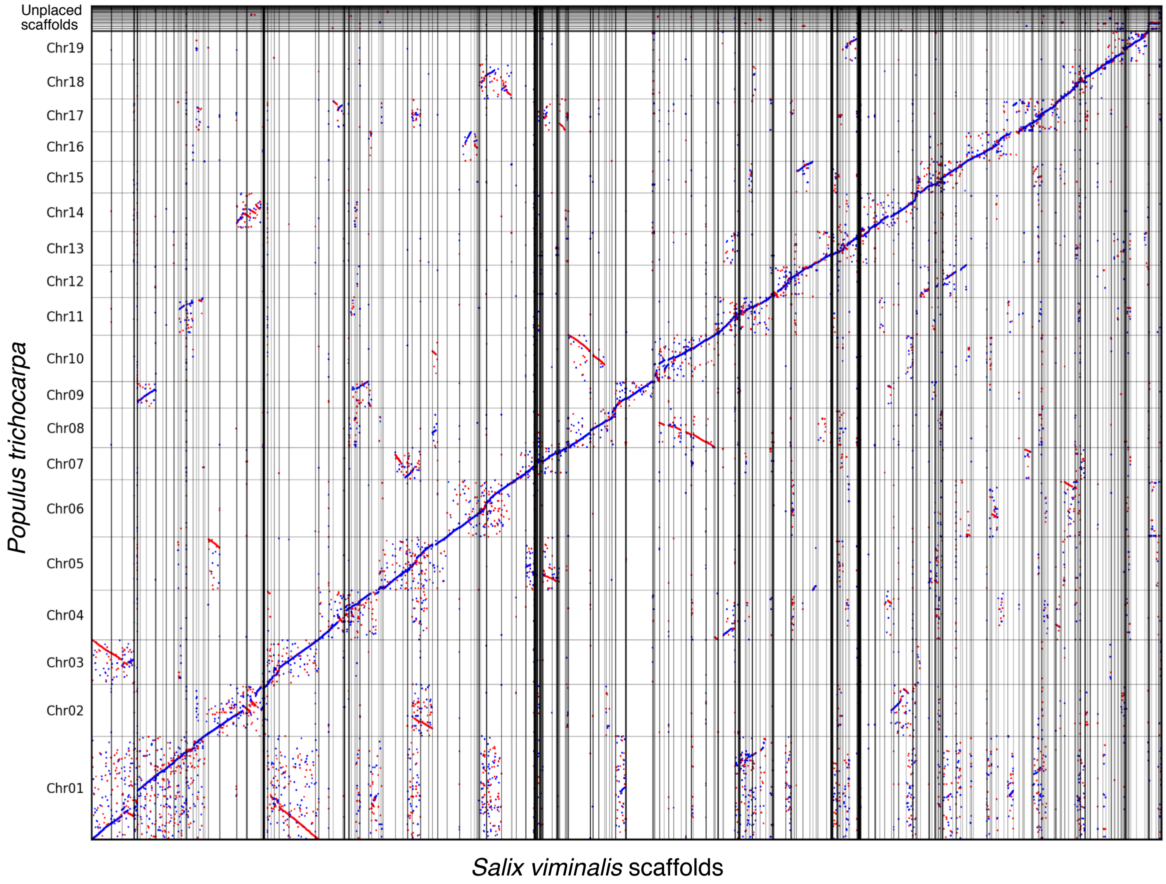

Supplemental Figure S2. Genetic markers aligned to chromosome 15 (from Pucholt et al. 2015) on our assembly. Markers associated with female segregation are filled in black. The grey shaded area delineates the location of the sex determining region (SDR) between 54.5 cM and 62 cM identified in Pucholt et al. 2017.

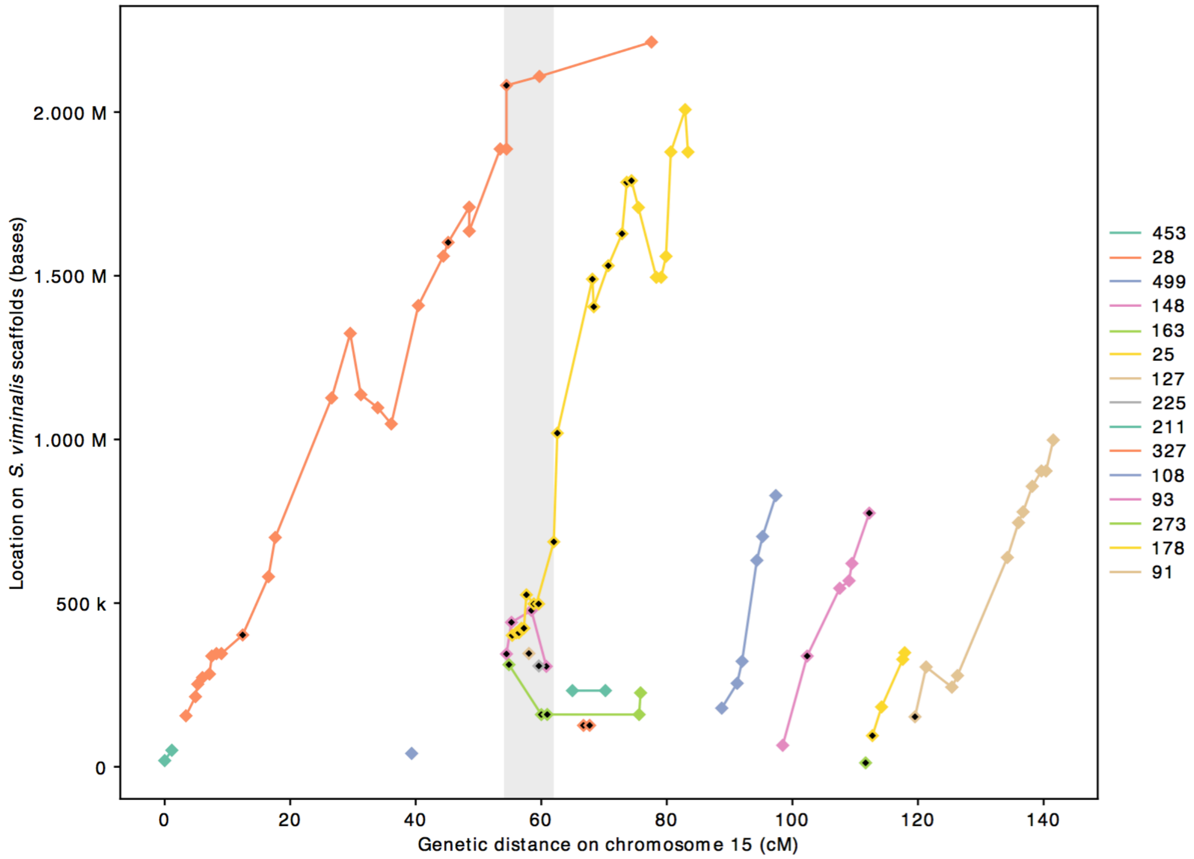

Supplemental Figure S3. Percentage of fully phased haplotypes using 10X Genomic Chromium sequence data, across the whole genome (**A**) and chromosome 15 (**B**).

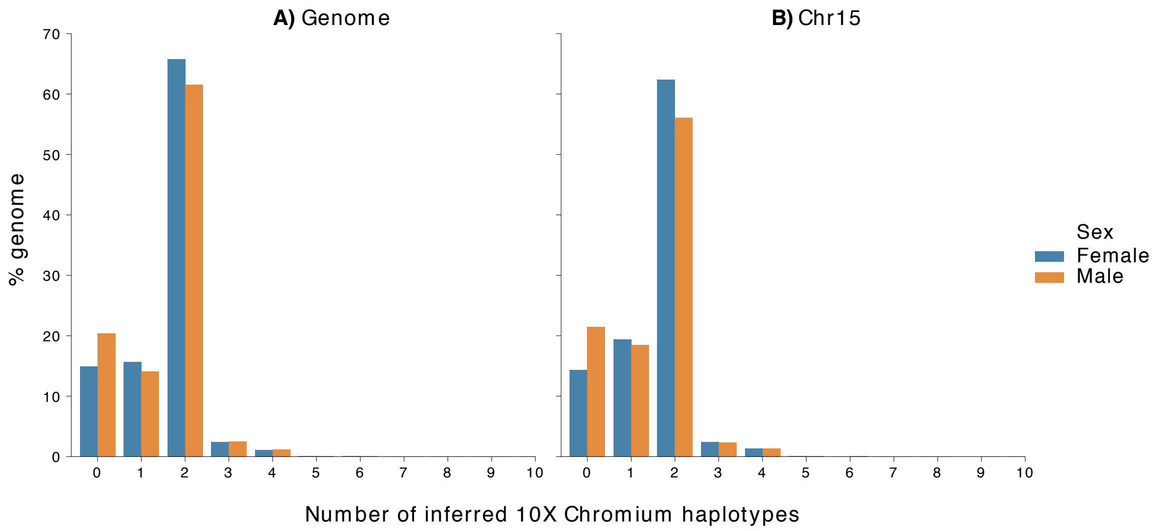

Supplemental Figure S4. Phylogenetic trees between Z-W gene pairs in the basket willow SDR. Female *Salix viminalis* haplotypes are indicated with red squares and male haplotypes with blue squares. Trees were estimated by maximum likelihood. Bootstrap values >75% are indicated with black dots on the respective nodes. The poplar (*Populus trichocarpa*) ortholog was used to root the trees. The name of each gene is indicated at the top of the tree and the scaffold where the gene is located is indicated in parenthesis.

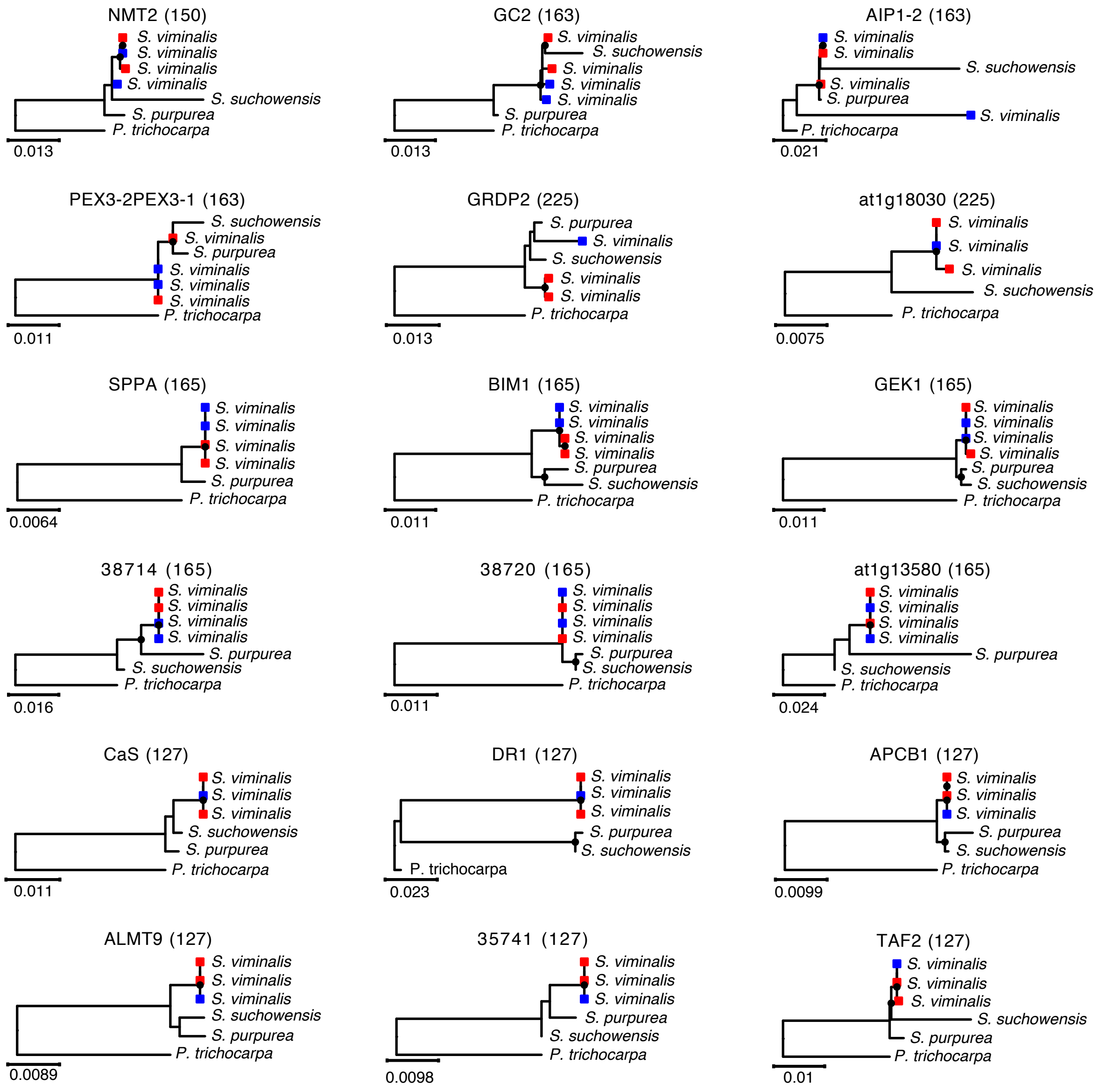

Supplemental Figure 5. Density of repetitive elements across different genomic regions.

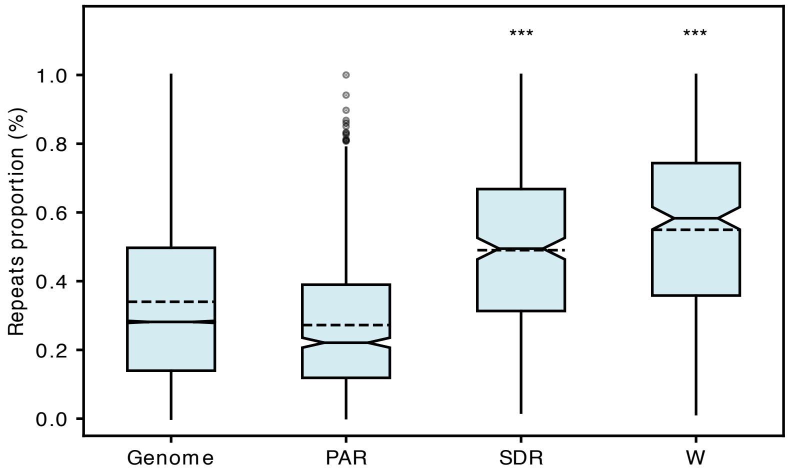

Supplement: Supplementary file 1 — Additional file 1: Table S1. Whole-genome DNA sequencing data used in this study. Table S2. Assembly statistics for the full and non-redundant assemblies of Salix viminalis. Table S3. Characterization of the different annotation classes for the Salix viminalis assembly. Table S4. Characterization of heterozygous structural variations identified in Chr15. Table S5. List of all genes found on putatively W-linked scaffolds. Figure S1. Whole genome synteny between Salix viminalis assembly and Populus trichocarpa. Figure S2. Genetic markers aligned to chromosome 15 (from Pucholt et al. 2015) on our assembly. Figure S3. Percentage of fully phased haplotypes using 10X Genomic Chromium sequence data. Figure S4. Phylogenetic trees between Z-W gene pairs in the basket willow SDR. Figure S5. Density of repetitive elements across different genomic regions. [file 12915_2020_808_MOESM1_ESM.pdf]
